# Supplementary material for: Disruption of Cytosolic Folate Integrity Aggravates Resistance to Epidermal Growth Factor Receptor Tyrosine Kinase Inhibitors and Modulates Metastatic Properties in Non-Small-Cell Lung Cancer Cells
Source: Int J Mol Sci. 2021 Aug 17;22(16):8838. doi: 10.3390/ijms22168838 (PMC8396212; doi:10.3390/ijms22168838)
Supplement: Supplementary file 1 [file ijms-22-08838-s001.zip › ijms-1292977-supplementary.pdf]

Supplementary Table S1. Primer sets used for qPCR

Gene	Forward Primer	Reverse Primer
MMP-1	5′-AGCTAGCTCAGGATGACATTGATG-3′	5′-GCCGATGGGCTGGACAG-3′
MMP-2	5′-GGCCCTGTCACTCCTGAGAT-3′	5′-GGCATCCAGGTTATCGGGGA-3′
MMP-7	5′-CTGGACGGATGGTAGCA-3′	5′-AAGAATGGCCAAGTTCATGAG-3′
MMP-9	5′-TTGACAGCGACAAGAAGTGG-3′	5′-TCACGTCGTCCTTATGCAAG-3′
NF-κB *(RELA)*	5′-GGCCATGGACGAACTGTTCCC-3′	5′-GGAGGGTCCTTGGTGACCAG-3′
SLUG	5′-TGGTTGCTTCAAGGACACAT-3′	5′-GTTGCAGTGAGGGCAAGAA-3′
SOX-9	5′-GTACCCGCACTTGCACAAC-3′	5′-TCGGTCTCGTTCAGAAGTCTC-3′
18S	5′-TGGCTCATTAAATCAGTTATG-3′	5′-CGGCATGTATTAGCTCTA-3′

Supplementary Figure S1

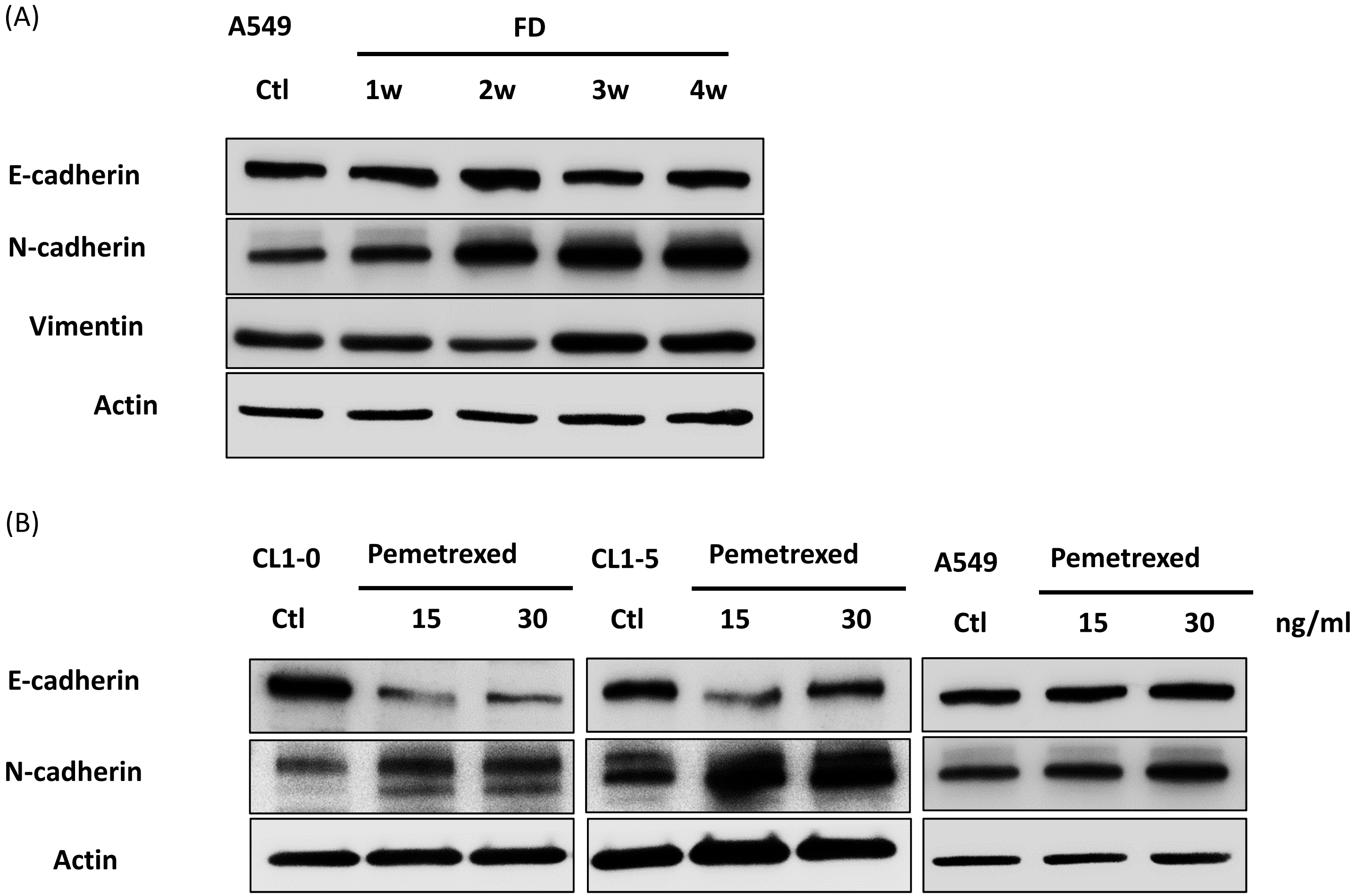

(C)

A549

A549

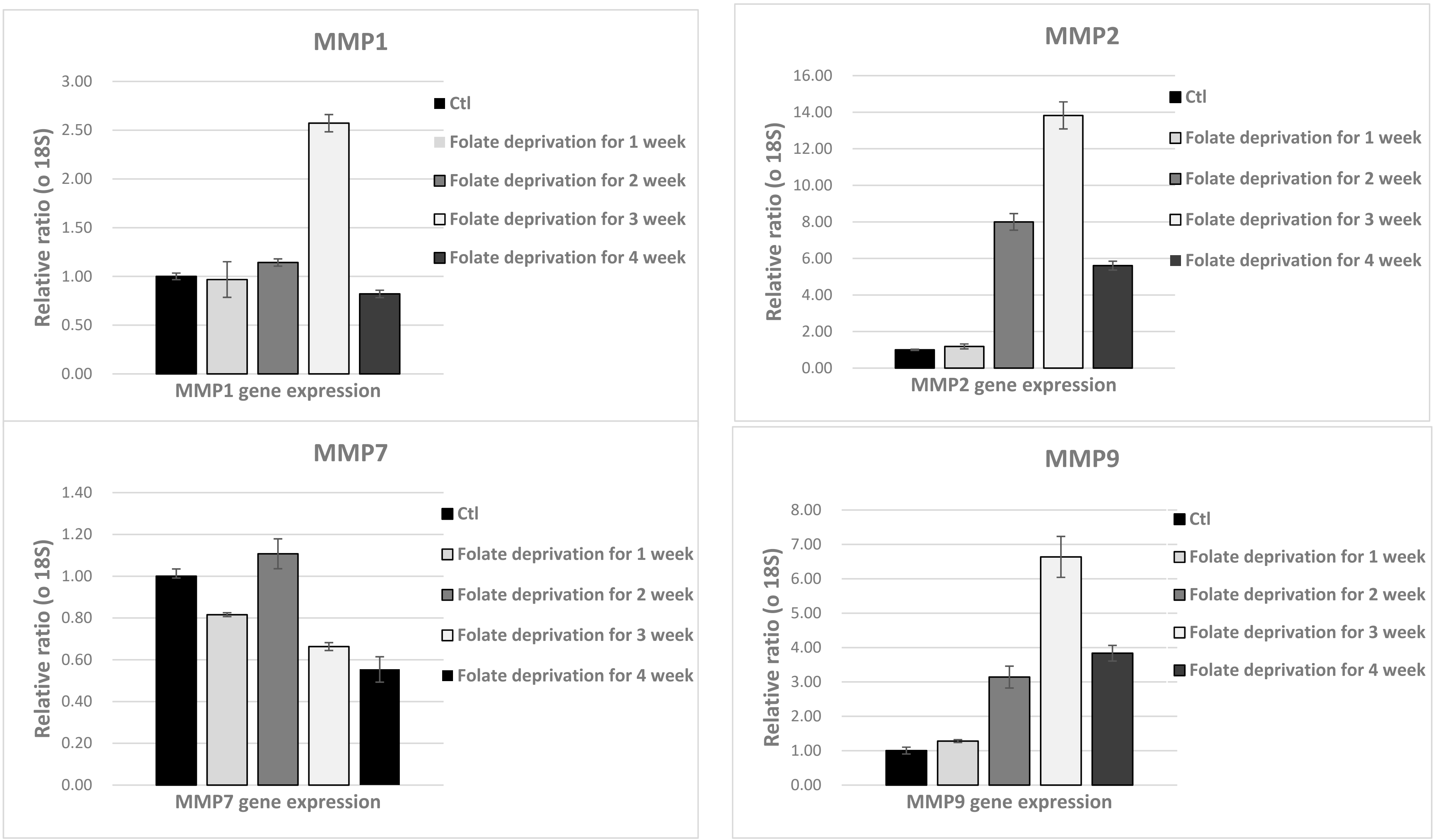
MMP1
3.00
2.50
2.00
1.50
1.00
0.50
0.00
Relative ratio (o 18S)
MMP1 gene expression
Ctl
Folate deprivation for 1 week
Folate deprivation for 2 week
Folate deprivation for 3 week
Folate deprivation for 4 week
MMP2
16.00
14.00
12.00
10.00
8.00
6.00
4.00
2.00
0.00
Relative ratio (o 18S)
MMP2 gene expression
Ctl
Folate deprivation for 1 week
Folate deprivation for 2 week
Folate deprivation for 3 week
Folate deprivation for 4 week
MMP7
1.40
1.20
1.00
0.80
0.60
0.40
0.20
0.00
Relative ratio (o 18S)
MMP7 gene expression
Ctl
Folate deprivation for 1 week
Folate deprivation for 2 week
Folate deprivation for 3 week
Folate deprivation for 4 week
MMP9
8.00
7.00
6.00
5.00
4.00
3.00
2.00
1.00
0.00
Relative ratio (o 18S)
MMP9 gene expression
Ctl
Folate deprivation for 1 week
Folate deprivation for 2 week
Folate deprivation for 3 week
Folate deprivation for 4 week
